# Green’s Improved Burr Engine

**Published:** 1872-04

**Authors:** WM. Taft


### Green's Pneumatic Burring Engine Improved.

BY WM. TAFT.

At the last annual meeting of the Mississippi Valley Dental Association there was on exhibition one of the above indispensable accessories to operative dentistry, as improved by Dr. E. R. E. Carpenter, of Chicago, who has the entire control of their manufacture and sale. The changes from the original instrument are of such importance that a knowledge of them must prove valuable to the profession.

The apparatus in all its parts has been increased in size one-third or more. The bellows, aside from this, is constructed of better frame and the finest calf skin, making it very durable, diminishing its liability to get out of order, and giving great additional motive power.

The machinery that carries the burrs, polishing buffs, etc., is superb in its construction, the metals used being of the highest standard, and heavily plated.

The revolving wheels are larger, consequently heavier, their shafts running in journals, enclosed in oil chambers, doing away with the necessity of lubricating every day or two. There is still further improvement in this direction under process of manufacture, consisting of jewels for the journals, the same as in watches.

The pinion is of smaller circumference—decreasing the speed and increasing the power. The burr shaft has a bearing of over half an inch.

The most serious objection to these instruments has been overcome, the want of power, it being now almost impossible to bear upon the burr with sufficient force to stop its revolutions. No force that can be used upon a tooth with safety will have any appreciable effect.

Another objection, serious to some, annoying to all, has been obviated, the catching of the beard and pulling out the hairs. The ladies need go to no expense for this luxury. Arrangements are being perfected by which the power can be supplied from outside the operating room, relieving the operator from extra exertion.

All who are acquainted with Dr. Carpenter are well aware that he knows a good thing, and with him to know is to do, and he is determined to keep his little buzz in the advance.

